# Effects of Tourist and Researcher Presence on Fecal Glucocorticoid Metabolite Levels in Wild, Habituated Sulawesi Crested Macaques (*Macaca nigra)*

**DOI:** 10.3390/ani13182842

**Published:** 2023-09-07

**Authors:** Dominique A. Bertrand, Carol M. Berman, Michael Heistermann, Muhammad Agil, Uni Sutiah, Antje Engelhardt

**Affiliations:** 1 Department of Anthropology, University at Buffalo, Buffalo, NY 14261, USA; 2 Evolution, Ecology, & Behavior Program, Department of Environment and Sustainability, University at Buffalo, Buffalo, NY 14260, USA; 3 German Primate Centre, Endocrinology Laboratory, Leibniz Institute for Primate Research, 37077 Goettingen, Germany; 4 Faculty of Veterinary Medicine, Bogor Agricultural University, Bogor 16680, Indonesia; 5 School of Natural Sciences and Psychology, Liverpool John Moores University, Liverpool L3 3AF, UK

**Keywords:** cortisol, wildlife tourism, primates, *M. nigra*, fecal glucocorticoids, ecotourism, stress

## Abstract

**Simple Summary:**

Ecotourism managers and field researchers often assume that primates residing in ecotourism locations are accustomed to people and therefore are not adversely affected by visitors. We examined the effects of tourist and researcher presence on three groups of critically endangered, wild crested macaques (*Macaca nigra*) in Tangkoko Nature Reserve, Sulawesi, Indonesia. Tourists visiting Tangkoko have access to macaque groups and can walk into them. Hence, we examined the possible effects of tourists both (1) in the reserve when outside and away from the study groups and (2) within study groups. We analyzed fecal cortisol metabolite levels (FGCM—a hormone that is often elevated when individuals experience stress) from 456 fecal samples (collected from thirty-three adults). When tourists were present in the forest, but not directly among the macaques, FGCM levels in the macaque groups were higher in months with greater tourist numbers. When tourists were within groups, some females displayed FGCM responses typical of acute stress. Male FGCM levels increased with numbers of tourists within the group, but did not change postexposure. FGCM responses to researchers varied by group, sex, and tourist presence. However, the temporal patterning of FGCM responses indicated little evidence of chronic stress from tourism at Tangkoko Nature Reserve.

**Abstract:**

Ecotourism managers and researchers often assume that apparently habituated primate groups no longer experience adverse consequences of prolonged exposure to tourists or researchers. We examined the effects of tourists and researchers on fecal glucocorticoid metabolite output (FGCM) in three critically endangered, wild crested macaque (*Macaca nigra*) groups in Tangkoko Nature Reserve, Sulawesi, Indonesia. We assayed FGCM from 456 fecal samples collected from thirty-three adults. Tourists can walk through and among macaque groups freely. Hence, we examined the possible effects of tourists both (1) in the reserve when away and not interacting with the study groups and (2) when they were present within the macaque groups. Generalized Linear Mixed Model (GLMM) analysis indicated that when tourists were present in the forest, but not directly among the macaques, FGCM levels in the macaque tourism groups were higher in months with more tourists. When tourists were among the macaque groups, some female macaques experienced rises and subsequent postexposure decreases in FGCM levels, consistent with predictions for acute stress. Male FGCM levels increased with tourist numbers within the group. Nevertheless, they were not significantly different from levels during undisturbed or postexposure conditions. FGCM responses related to researchers in groups varied by group, sex, and tourist presence. However, the temporal patterning of FGCM responses showed little evidence of chronic stress from tourism at this site.

## 1. Introduction

The concept of ecotourism stems from the belief that environmentally responsible tourism can both be a financial boon to local populations and help conserve the environment [1,2]. However, many ecotourism operations have been shown to damage local wildlife through anthropogenic disturbances such as increased pollution [3], disruption of daily wildlife routines and social behavior [4], increased risk of disease transmission [5,6,7], and indications of increased stress in wildlife [8,9,10,11]. These risks are more concerning at sites that feature primates, whose conservation status is often considered as an indicator of the overall health of their ecosystems [12]. However, while practices in many ecotourism locations are designed to reduce potential harm from anthropogenic factors, managers and researchers at sites in operation for decades often do not consider measuring potential increases in primate stress as a need [13]; see also [14] for review. Often, this is because they assume that, due to decades of apparent habituation (i.e., cessation of behavioral responses to a once-novel situation), primates targeted for tourism no longer experience harmful consequences of prolonged exposure to tourists or to the researchers [15]. Unfortunately, this assumption is risky given evidence that unavoidable, chronic exposure of primates to humans can be associated with behavioral and physiological manifestations of stress [11,16,17]. Increased research into behavioral and physiological responses of individual primates to tourism, while carefully controlling for potentially confounding factors, can help identify ecotourism practices that need modification.

### 1.1. Stress Physiology—Acute vs. Chronic

Organisms maintain an internal equilibrium (homeostasis) through coordinated physiological responses to stimuli that are perceived to threaten their normal function (i.e., stressors) [18]. Acute stressors activate a cascade of physiological responses [19], including the secretion of glucocorticoids (cortisol or corticosterone) [20]. These physiological responses can be adaptive by meeting short-term increases in metabolic demand needed for an effective “fight or flight” response [21]. When an animal is not experiencing a stressor, it usually displays relatively low glucocorticoid levels. Normal responses to acute stressors (i.e., acute stress) generally involve short-term increases in glucocorticoids within a few hours for salivary levels (e.g., within 2 h: *Ovis aries*, [22]; ~2 h: *Pan Paniscus,* [23]); within 24 h for urinary levels: *Macaca fascicularis*, *Pan troglodytes* [24]; and within a few days for fecal levels (e.g., ~36 h: *Macaca nigra* [25] or ~48–72 h: *Pongo pygmaeus morio*, [26]). Fecal glucocorticoid levels then return to undisturbed levels quickly after the stressor is no longer perceived (*P.p. morio*: [26]; *M. fasicularis*, *P. troglodytes:* [24]; and *M. nigra:* [25]).

However, when an organism experiences stressors for extended durations (i.e., chronic stress), the prolonged release of cortisol is energetically expensive and disruptive to other physiological processes, including immune function, reproduction, and growth [21]. Chronic stress responses in primates are marked by high baseline glucocorticoid levels with no or minimal increases when exposed to an acute stressor and/or a delay in the return to undisturbed output levels following the end of a stressor [27,28].

While the harmful effects of chronic stress on health and fitness are well documented, researchers are still uncovering the effects of frequent acute glucocorticoid responses on health and fitness. For example, frequent acute physiological stress can potentially disrupt reproductive endocrine processes and affect fertility [29,30,31,32,33].

### 1.2. Measuring Physiological Stress in Wildlife

In this study, we asked whether glucocorticoid levels were affected by tourism in wild, apparently habituated Sulawesi black crested macaques (*M. nigra)* in the Tangkoko Nature Reserve by measuring fecal glucocorticoid metabolites (FGCMs) as a proxy for the physiological stress response. Currently, the least invasive way to measure glucocorticoid levels in wildlife is through feces [34]; see also [35] for review. Cortisol itself is generally not present in feces; depending on the species, it breaks down into several metabolites. FGCM can indicate average cortisol levels over a day. In many mammals, including several primates, glucocorticoid metabolites in feces peak about 24–48 h after a stressful event is no longer perceived [25,36,37] and return to undisturbed levels about 48–72 h after the peak [24,26,37].

Properly timing the collection of samples can help uncover whether responses are consistent with acute or chronic stress responses to anthropogenic stress. It is also important to control for other influences on glucocorticoid levels. Notably, higher-than-normal glucocorticoid levels indicate that demands for energy expenditure and mobilization are high, and that may be due to any number of factors [38]. In addition to real or perceived risks of tourism, FGCM levels in our macaque study groups could increase with environmental pressures, e.g., food resources [39], extreme weather [40,41], reproductive hormones (estradiol: [33,42,43]; testosterone: [44,45]), vigorous physical activity [46] and others. Thus, researchers can use measurements of FGCM levels to shed light on sources of anthropogenic stress in ecotourist sites provided they control for other factors that potentially increase metabolic demand.

### 1.3. Evidence of Physiological Stress Related to Primate Ecotourism

A recent meta-analysis of anthropogenic impacts on physiological stress in wild primates by Kaisin et al. [16] found that primates living in sites experiencing various anthropogenic disturbances exhibited higher glucocorticoid levels than those who were not. However, they saw this only under the conditions of habitat loss and hunting. While they did not find an overall significant effect when looking at tourism, there are several examples from the literature that indicate increases in physiological stress related to tourism. For example, wild *P.p. morio* showed an increase in FGCM following tourist visits [26]. Additionally, Shutt et al. [11] found that two Western lowland gorilla (*Gorilla gorilla gorilla*) groups that were apparently habituated to tourism (one recently and one for a longer period of time) had higher FGCM levels than a non-human-contacted unhabituated group. Also, in these same tourism-designated groups, when tourists violated the “no closer than 7 m to the group” rule, *G.g. gorilla* FGCM levels increased. Barbary macaques (*Macaca sylvanus*) also had higher fecal glucocorticoids after aggressive interactions with tourists [47]. At this time, it is not clear whether the inconsistencies in findings among studies (e.g., [16] vs. [11,26,47]) are due to species differences, the lack of control for other factors that may affect metabolic demand as described above, or other issues. As such, they highlight the need to explore physiological stress responses to tourism using a variety of primate species and circumstances while controlling for possibly confounding factors.

### 1.4. Macaca Nigra and Tourism in Tangkoko Nature Reserve

One species in which such questions can be ideally examined is the Sulawesi black crested macaque, *M. nigra*, living in Northeast Sulawesi, Indonesia. Black crested macaque social organization is female-philopatric and female-bonded [48] with nonseasonal breeding, but with a tendency toward birth peaks between January and May [49]. The macaques use a variety of habitats, subsist primarily on fruit supplemented with plant parts as well as invertebrate and vertebrate prey [50], and live in large multimale, multifemale groups. They are diurnal and semiterrestrial, spending 59% of their day traveling, foraging, and feeding, with the remaining time spent resting and socializing [51]. Male crested macaques disperse at sexual maturity and secondarily at intervals throughout adulthood [52]. For additional details on *M. nigra* and current status, see [53,54].

The Indonesian government and local villagers of Batu Putih jointly manage ecotourism in Tangkoko Nature Reserve (TNR). Local villagers earn income directly from the government by serving as park employees, rangers, and firefighters. Tourists also provide income by paying for lodging, food, and guides. In the last 25 years, the number of inns in the village has grown from one to eight, with more planned and several restaurants in the works. Prior to the COVID pandemic, many Batu Putih citizens used tourism as their primary source of income, and fewer took jobs in the nearby gold mines (personal communication with local people, 2017). With Indonesia opening back up to visitors, tourists are returning to TNR (D.B., personal communication with local people, 2023).

For over three decades, unfamiliar humans (tourists) have visited several crested macaque social groups almost daily. Park rules constantly change, but in general, guides must remain with tourists while they are inside TNR, unless tourists wish to go to the beach. Tourist groups range in size from 2 to 25 individuals (with cruise ship tour groups and school groups increasing this to 100). Although trails exist to help traverse the forest, tourists do not need to remain on them. They explore the forest from dawn until dusk and can walk into a group of macaques unhindered. Although a “research-only” zone exists to protect some macaque groups from frequent tourist visits, guides sometimes lead tourist groups to a giant strangler fig in the center of this zone. Along the way, tourist groups may occasionally encounter a macaque group that is designated for research only, in which case researchers within the Macaca Nigra Project (MNP—our research site manager and research sponsor) can request that they leave the area as quickly and quietly as possible. Macaque groups less habituated to tourism also tend to move farther away from or actively avoid tourists (D.B., personal observation).

Two distinct *M. nigra* groups most often occupy the tourist zone. During the low tourist (rainy) season, these macaque groups are exposed to an average of two tourist groups per day. During the high tourist (dry) season, they can be exposed to as many as seven tourist groups per day, often more than one at a time. The closer a macaque group is to the main entrance, the more likely it is to encounter tourists. Park rules prohibit feeding and interacting with macaques and discourage flash photography; however, guides rarely enforce these rules and sometimes actively encourage feeding and touching of the macaques.

### 1.5. Other Anthropological Influences

Batu Putih villagers keep gardens directly abutting the reserve that have historically been a sought-after food source for *M. nigra*. When MNP began research inside TNR, the program directors recognized the importance of employing someone whose sole job was to defend the village against crop foraging using nonlethal methods. Any macaque group that ranges close to the village border experiences nonlethal crop defense to encourage them to return deeper into the TNR.

Prior research examined the effects of tourism on crested macaque behavior and physiology inside TNR, but none have investigated physiological responses on an individual level. Paulsen [55] found that crested macaques displayed aggressive behaviors more frequently and escalated aggression more quickly when in the presence of tourists, and also that fecal cortisol metabolite levels (pooled by group) were lower in a group experiencing intermediate numbers of tourists than either a group experiencing more tourists or a group experiencing almost no tourism. More recently, Bertrand et al. [56] found that *M. nigra* groups displayed behavioral changes when exposed to tourists. Specifically, macaques showed signs of behavioral inhibition (a general decrease in several behaviors indicative of vigilance) when more tourists were present in the forest. They also showed signs of both inhibition and increases in stress related behavior (for example aggression) when tourists were directly present in groups, similar to how primates perceive predators posing varying degrees of risk.

**Figure 1:**
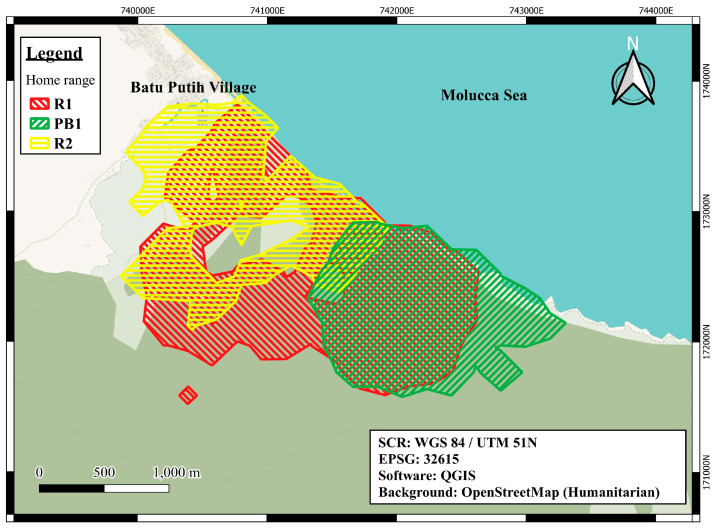
*Macaca nigra* home ranges inside Tangkoko Nature Reserve from Oct 2015 to Jan 2016. From Dr. Laura Martinez-Inigo. Copyright Dr. Laura Martinez-Inigo, 2023 with permission. The home ranges are the 95% isopleths of the Brownian Bridge Movement Models. The polygons roughly represent where the macaques were 95% of the time.

Here, we test the general hypothesis that levels of FGCM are associated with aspects of tourism in three apparently habituated groups of wild *M. nigra* in TNR named R1, R2, and PB1. These habituated groups represent a natural experiment, each exposed to different frequencies of tourist visits (study group names: R2 = frequently, R1 = moderately, and PB1 = rarely/research only). The natural experiment is due to their ranging patterns (see Methods, Section 2.2, Figure 1); R2 was visited by tourist groups almost daily, R2 was visited generally 3–5 days a week, and PB1 generally once every other month.

### 1.6. Specific Hypotheses (Table 1)

Although one study group (PB1) rarely encountered tourists, tourists can be loud. Large groups of tourists can be heard by all three study groups from far away. For this reason, we explored the possible effects on macaque FGCM levels of (1) tourists in the reserve when away from the study groups and (2) tourists while in the presence of the study groups.

## 2. Materials and Methods

### 2.1. Study Site

The study site, Tangkoko Nature Reserve (TNR), is an 8867-hectare nature preserve located in NE Sulawesi, Indonesia. TNR is likely home to the last remaining, natural, and viable population of Sulawesi black crested macaques (*M. nigra)*. A large introduced population exists on the nearby island of Bacan [57,58].

### 2.2. Study Groups

Our three study groups were: R2 (22–23 adults), R1 (40–42 adults), and PB1 (22–23 adults). While all group ranges overlapped and included spots commonly used by tourists, R2’s range was closest to the village and the park entrance, R1’s range was at an intermediate distance, and PB1’s range was the farthest of the three (Figure 1). The two tourism groups (R1 and R2) have been exposed to tourists (and accompanying guides) for approximately two and three decades, respectively. To date, park rangers have not limited the daily number of guides or tourists. TNR tour guide rules (unpublished, distributed in 2015) do state that each guide is limited to four tourists. However, we frequently saw more guides or tourists in macaque groups. MNP limited the numbers of researchers per group to six for R2 and R1 and four for PB1. Finally, the study groups were also exposed to crop defense when they ventured close to the park boundaries. This occurred frequently with R2, moderately with R1, and rarely with PB1. Thus, we controlled for the frequency of crop defense in our statistical analysis.

### 2.3. Subjects

Our subjects were 33 adult *M. nigra* (age ≥ 7 years, 15 males and 18 females; Table S1). When possible, we selected macaques to be comparable to each other across study groups. Females were ranked as low, middle, or high and categorized by age as young: 7–10 yrs, middle: 10–13 yrs, or old: >13 yrs. We selected only young or middle-aged females, two from each rank category. We were unable to implement this with males because PB1 and R2 had fewer than six males each. MNP provided previously determined macaque ranks (David scores for females and Elo ratings for males) and macaque age categories to us before our data collection period started. Changes were anticipated in male ranks because they were highly asymmetrical and linear [52]. Thus, we tracked changes in male dominance rank over time using Elo rating (*cf* [59]). On the other hand, female *M. nigra* ranks were both linear and generally stable over time [48]. Hence, we used David’s Score to assess female rank changes throughout the study period. For both sexes, we used agonistic behaviors (see definitions in Table S2) for an assessment of rank relationships.

We followed four males and six females from PB1, six males and six females from R1, and five males and six females from R2. No adult males migrated into the group during the study period. Some of our initially selected subjects (one male and three females) either died or disappeared during our study, and were not included in the analysis. We could not replace the male but were able to replace the females with subjects of comparable age and rank.

### 2.4. Field Methods

From October 2014 to January 2016, DB and six assistants collected fecal samples and recorded an ethogram of behaviors (see Table S2), including locomotion. All team members participated in interobserver reliability testing for identity recognition, using long-term observers (Research Manager and permanent field assistants) as standards. We tested macaque IDs until we could identify 100% of the macaques in each group. Interobserver reliability scores for locomotion also reached high levels (kappa coefficient = 96%). We also recorded the number of guides, tourists, researchers, and daily crop defense occurrences in each macaque group per day. As often as possible, team members went to each social group in teams of two. Group selection was as random as possible but depended on which group locations were known and whether other teams needed data from particular study groups that day. Data on average rainfall for each month of the year data were collected came from the weather site Weather Underground (http://www.wunderground.com (accessed on 15 March 2018)).

### 2.5. Physical Activity

As FGCM increases with metabolic demand, we collected physical activity data on an individual level to control for this possible confound. Our measure of physical activity was the mean percentage of time individuals spent locomoting (walking, running, and climbing) per month, which we calculated from point time samples taken at the start of 2 min focal animal sampling sessions.

### 2.6. Physiological Data Collection

The team collected fresh fecal droppings noninvasively immediately after defecation, once a day, from as many focal macaques as possible. We collected samples from the same individuals on as many consecutive days as possible to maximize the chances that samples would be available for specific tourism conditions. Given that all data collectors were able to identify individual macaques with 100% accuracy (see above), we were confident about the identity of the macaque that deposited each fecal sample. We used only solid (not watery) fresh stool (0.5 g) that was uncontaminated with urine from the identified macaques. Although *M. nigra* do not display significant diurnal variation in levels of fecal cortisol metabolites [60], we recorded the time collected. Samples representing undisturbed conditions were collected after two full days with no tourists or crop defense events. The timing of samples representing exposure to tourists represented some special issues because the tourism groups R1 and R2 frequently experienced consecutive days (2–36 days) of exposure to either tourists, crop defense, or both. Means ± SD for consecutive days with tourists and/or crop defense were 2.70 ± 2.26 for R1 and 6.94 ± 8.03 for R2. To minimize potential variation in effects due to long and varying-length runs of exposure, we used fecal samples collected the day after only 1–4 consecutive days of exposure to tourists. We chose four as a limit based on perusal of the distribution of FGCM levels by number of consecutive days of exposure and found a discontinuity in the distribution of FGCM levels between 4 and 5 days (*n* = 477). This resulted in a set of samples taken a mean of 36–48 h after the onset of exposure to tourists in each run of days, an interval in line with peak response times to natural stressors in this population [25]. Samples representing postexposure conditions were collected two days after the final day of a run of exposure to tourists and when there were no intervening days of exposure to tourists or crop defense. Unfortunately, tourist visits and crop defense events were so frequent at Tangkoko Nature Reserve that the timing of postexposure samples was not ideal. Mean ± SD consecutive days between runs of tourists and/or crop defense events were 1.52 ± 1.07 for R1 and 2.22 ± 1.57 for R2). Thus, in most cases, there were few days between runs of days with tourists or crop defense events such that it was not possible to collect samples representing longer postexposure intervals. Hence, we were able to detect quick returns to undisturbed levels of FGCM, but we could not precisely measure the length of slower returns.

After collection, researchers placed the sample on a large leaf for processing and removed all indigestible elements (seeds, grass, and leaves), mixed the samples thoroughly [61], and added 0.5 g to a graduated, twist cap collection tube filled with 5 mL of 80% ethanol. They then shook the tube by hand for 30 s to create a fecal ethanolic suspension [62]. The samples were stored in the dark at ambient temperatures in the field until researchers returned to the field station at night. For the extraction of FGCMs carried out directly after return to the field camp, we used a validated and proven field-friendly extraction method (e.g., [62,63,64,65,66]). In brief, we extracted FGCMs by shaking the fecal suspension manually for 2 min and then centrifuging it using a manually operated centrifuge [63,66]. The individual extracts were then decanted into two 2 mL snap cap storage tubes, sealed with Parafilm, and stored in a freezer [36] until transport to the endocrinology laboratory of the German Primate Center for FGCM analysis. We sun-dried the remaining fecal pellets following extraction [66] to a constant weight in order to determine the dry weight of each sample. This weight was used for calculating final FGCM concentrations as ng/g dry fecal weight.

In total, we collected 546 fecal samples. We also used fecal samples collected for other studies by Dr. Celine Bret and Dr. Lisa M. Danish from our study groups during the project period. All sample collection and storage methods used matched those for our study. This increased our fecal sample size to 950 samples. After discarding samples taken after runs of more than four consecutive days of exposure to tourists (see above), 477 samples remained for FGCM analysis (Table 2).

### 2.7. Phenology and FAI

When food is scarce, cortisol is produced to metabolize stored energy reserves [21]. Due to this, low food availability, particularly fruit sugar [67], is sometimes associated with higher fecal glucocorticoid levels [68,69,70] and increased glucose mobilization. For this reason, we controlled for food availability by using phenology data to calculate a food availability index (*cf* [71]) (see below). Phenology data were collected monthly by MNP using a method designed by Dr. Oliver Schülke (see [56] for methodological details). We controlled for food availability even though we did not expect these factors to be major confounds in TNR. First, total food availability inside the reserve fluctuated relatively little across the seasons, and figs, a primary fruit source, were present in all months [72]. Second, variations in monthly levels of food/fruit availability and consumption were not closely correlated with variations in monthly tourist numbers [Pearson’s r (9) = −1.14, *p* = 0.282] or the number of days in a month with crop defense events [Pearson’s r (9) = −0.12, *p* = 0.726]. The FAI formula is described in (Table 3).

### 2.8. Hormonal Analyses

Fecal extracts were analyzed for FGCM levels using a microtiter plate enzyme immunoassay (EIA) for immunoreactive 11ß-hydroxyetiocholanolone [73], a major metabolite of cortisol in the feces of many species of primates [37,74]. The assay, carried out as described by [29], has been validated for monitoring glucocorticoid output in numerous primate species [37,62,75,76], including the study species [25,77]. Intra- and interassay coefficients of variation of high- and low-value-quality controls were 6.4% and 8.0% (high) and 7.9% and 10.8% (low).

Fecal extracts of females were measured for levels of estrogens using a microtiter plate enzyme immunoassay for the measurement of conjugated estrone (E1C), an abundant estrogen in macaque feces [78,79]. The measurement of E1C, which was carried out as described elsewhere [80], has previously been validated to reliably reflect ovarian activity in female crested macaques [81]. Intra- and interassay coefficients of variation of high- and low-value-quality controls were 5.4% and 7.2% (high) and 6.3% and 7.7% (low).

Fecal extracts of males were measured for levels of immunoreactive androgen metabolites using a microtiter plate enzyme immunoassay for the measurement of epiandrosterone (EA), an abundant metabolite of testosterone in macaque feces [74,82]. The assay has been successfully applied to monitor male androgen status in numerous primate species [74,76,83,84] and has also been biologically validated for crested macaques [25]. Intra- and interassay coefficients of variation of high- and low-value-quality controls were 7.4% and 8.6% (high) and 9.3% and 14.3% (low).

### 2.9. Data Analysis

We tested the predictions of H1-H4 using Generalized Linear Mixed Model (GLMM) analysis from the LME4 package version 1.1-30 [85] in R Studio 1.4.1103/R 4.2.1 (2022-06-23) using a log link function. Because we were not always able to match fecal samples from consecutive days for a given focal subject, our statistical analysis used measures of FGCM that were lumped by tourist condition within each focal subject. As such, it was possible to identify the donor of each sample and to control for individual macaque IDs in each model.

We ran separate models for each major hypothesis and for each sex. See Table 4 for a list of specific model factors, including the response variable, predictors, controls, and random effects. To ensure all fixed and random effects were not highly correlated, we tested for collinearity among variables; Variance Inflation Factors (VIFs) for all fixed and random factors were below 3. These included frequencies of crop defense events per month (GVIF^^(1/(2*Df)^ range = 2.05–2.17) and frequencies of tourists visits per month (GVIF^^(1/(2*Df)^ range = 1.34–1.36). Thus, although total tourist numbers (R2 = 1420, R1 = 750, and PB1 = 28) and total frequencies of crop defense events (R2 = 140, R1 = 24, and PB1 = 2) in each group were concordant, it was possible to control for responses to crop defense. We identified the appropriate distribution family for GLMMs by exploring data visually with qnorm functions and statistically with Shapiro–Wilk tests. All response variables indicated Poisson distributions.

For H1–H3, our unit of analysis was the individual fecal sample. We started by running a null model and then ran a full factor model (null and full factor model R code in Table S3). To evaluate differences between the full and null models, we applied Bonferroni corrections separately to each model, setting a critical level of 0.05/5 = 0.01. All full models differed significantly from the null, indicating that one or more fixed effects in the full model were associated with variation in the response factor. The criteria for significance for fixed and interaction effects was *p* ≤ 0.05. We checked all models for overdispersion by testing whether the model deviation was larger than the mean. If a model was significantly overdispersed, this indicated greater variability (statistical dispersion) in a data set than expected based on a given Poisson statistic. In these cases, we corrected overdispersion by creating an additional random intercept for each focal session [86]. After each GLMM, we evaluated the fixed effects using a likelihood ratio test. The models for H4 followed the same methods as listed above. However, our unit of analysis was each macaque’s average undisturbed monthly FGCMs. After each GLMM, we evaluated the fixed effects using a likelihood ratio test. Finally, we calculated the residuals of the FGCMs with each respective hormone. We then used the residuals as the outcome variable in a linear mixed model, controlling for the number of fertile females in the group, sex, rank, group, monthly tourist numbers, monthly crop defense occurrences, number of infants in the group, and FAI, with macaque ID as a random factor.

## 3. Results

### 3.1. Possible Physiological Effects of Tourists in the Forest

**Figure 2:**
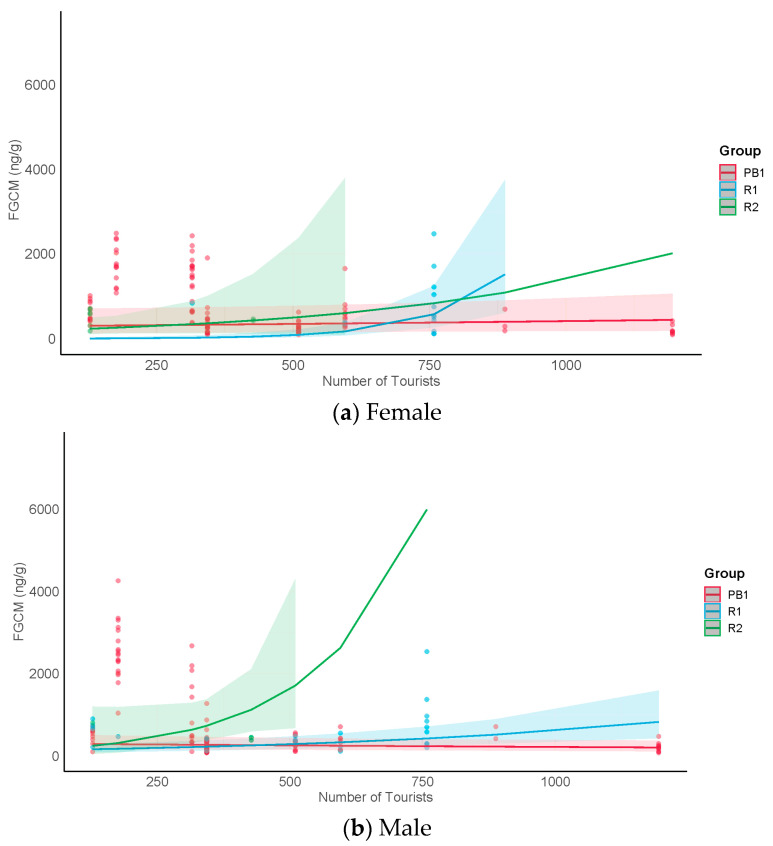
Undisturbed samples: group comparisons of female (**a**) and male (**b**) FGCM levels (ng/g) by numbers of tourists in the forest each month. Lines show predicted values for each group; shading shows 95% confidence intervals; dots show raw data points.

The results for H1, which examined FGCM levels from undisturbed samples (representing days with no tourists or crop defense within the group within the previous 48 h), are shown in Table 5. Although there were no significant main effects suggesting that FGCM levels were higher for either males or females in months when more tourists were in the forest (H1a), or that they were higher in the tourism groups (R1 and R2) than in the research-only group (PB1) (H1b), we found a significant interaction between monthly tourist numbers and group (H1c). We found that study groups and sexes responded in qualitatively different ways. The post hoc results (Table 5) suggest that female FGCM levels in the tourism groups also increased in the months with more tourists, but showed no change in the research-only group (Figure 2a). Male FGCM levels in the tourism groups increased with monthly tourist numbers, whereas those for the research-only group decreased (Figure 2b).

### 3.2. Physiological Effects of Tourists in the Group

**Figure 3:**
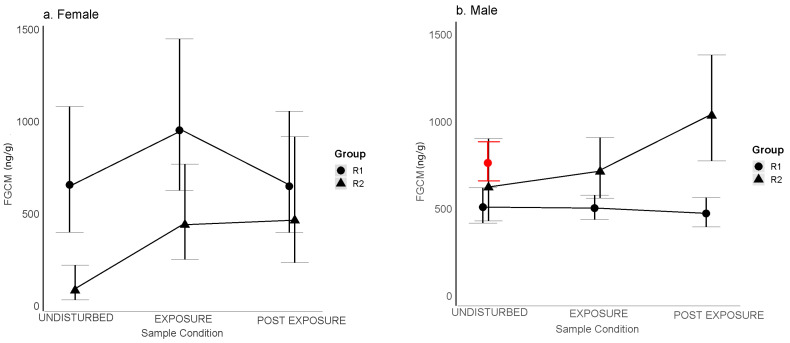
Female (**a**) and male (**b**) FGCM (ng/g) response to tourist presence during undisturbed, exposure, and postexposure tourism conditions. Red dot in (**b**) represents mean undisturbed level for PB1. Error bars show 95% confidence intervals.

**Figure 4:**
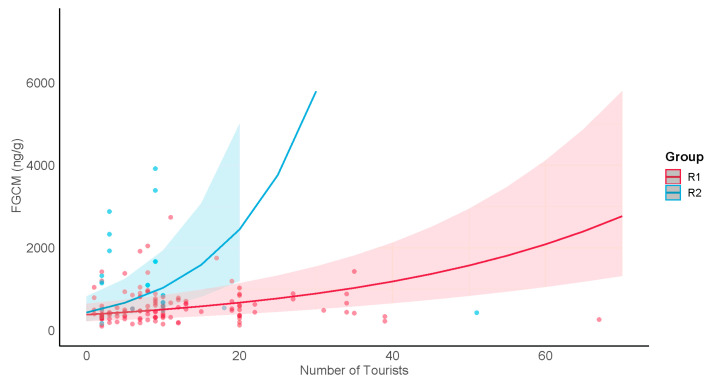
Male FGCM (ng/g) response to daily numbers of tourist present within the group. Lines show predicted values for each group; shading shows 95% confidence intervals; dots show raw data points.

The results for H2a–c, which compared FGCM levels representing conditions of undisturbed, exposure, and postexposure to tourists, and for H2d, which focused on FGCM levels representing the exposure condition, are shown in Table 6 and Table 7. In H2a, 2b, and 2c, we asked if patterns of metabolic demand in the presence of tourists would be consistent with those described for acute stress, for chronic stress, or the absence of stress, respectively. H2a was supported for R1 females but not for R2 females or for males. The FGCM levels for females differed significantly between exposure conditions. The post hoc results indicated significantly lower FGCM levels when undisturbed than during exposure, and a significant drop from exposure to postexposure levels (Figure 3a). R2 females also showed a significant increase from the undisturbed to the exposure samples, but no significance decrease from exposure to postexposure (Figure 3a). In contrast, the FGCM levels for R1 and R2 males showed no significant differences between exposure conditions (Figure 3b). However, the pattern of changes between conditions for R2 males appeared similar to that for R2 females. These results may be consistent with either predictions for chronic stress (H2b) or, in the case of males in both groups, the absence of a stress response (H2c). To distinguish between these two possibilities, we compared undisturbed levels of males in the tourism groups with those in the research-only group (PB1) (Figure 3b). We found that male undisturbed levels in the tourism groups were not significantly different from those for males in the research-only group (F = 2.50, *p* = 0.10) (Figure 3b), suggesting that male patterns of FGCM response were more consistent with a lack of a stress response than with chronic stress (see Discussion, Section 4). Finally, in H2d, we asked if FGCM levels were higher for exposure days in which more tourists were present within the group. Male FGCM levels in both groups increased, with R2 showing a more rapid rate of increase (Figure 4). Female FGCM levels showed no significant change (Figure S1).

### 3.3. Possible Effects of Researchers in the Group

**Figure 5:**
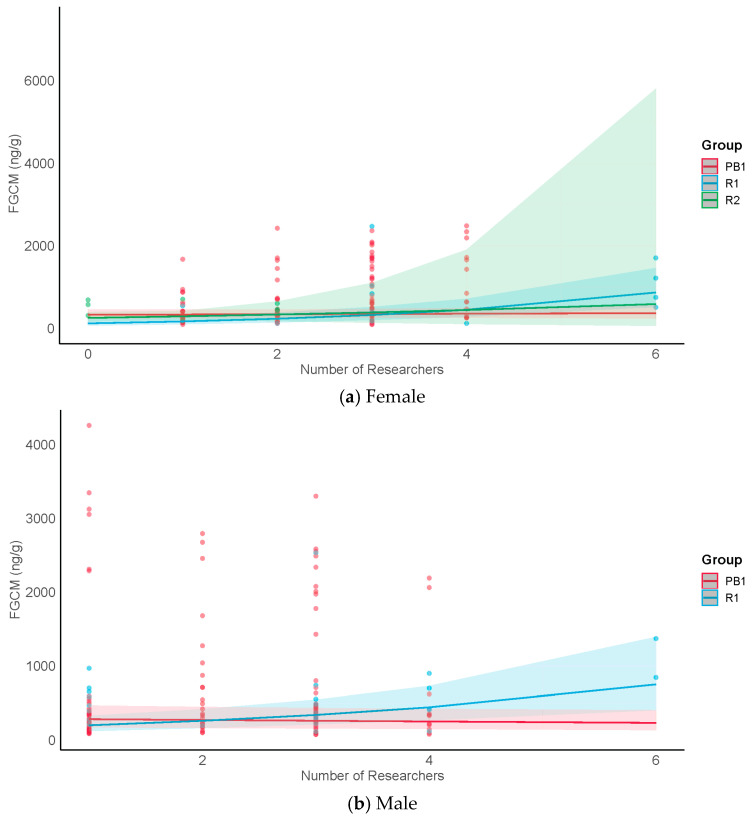
Undisturbed samples: group comparisons of female (**a**) and male (**b**) FGCM levels (ng/g) and numbers of researchers in the group each day. Lines show predicted values for each group; shading shows 95% confidence intervals; dots show raw data points.

The results for H3, which examined FGCM levels representing undisturbed (all three study groups) and tourism exposure (tourism groups R1 and R2) conditions as a function of the number of researchers present in the group, are shown in Table 8 and Table 9. Because the sample sizes were not adequate for R2 males in the undisturbed condition, the male undisturbed results apply only to R1 and PB1. The results varied based on tourism condition and sex. For the undisturbed condition, there were no main effects of researchers for either males or females. However, there were significant interactions between the numbers of researchers and group. Undisturbed FGCM levels increased as the number of researchers increased for R1 males, but decreased for PB1 males (Figure 5b). Undisturbed FGCM levels for R1 and PB1 females increased as the number of researchers increased with R1’s rate of increase greater than PB1’s (see Figure 5a). In contrast, R2 showed no significant change. The responses by PB1 to several (5–6) researchers were uncertain since there was a maximum of only 4 researchers allowed in this group.

**Figure 6:**
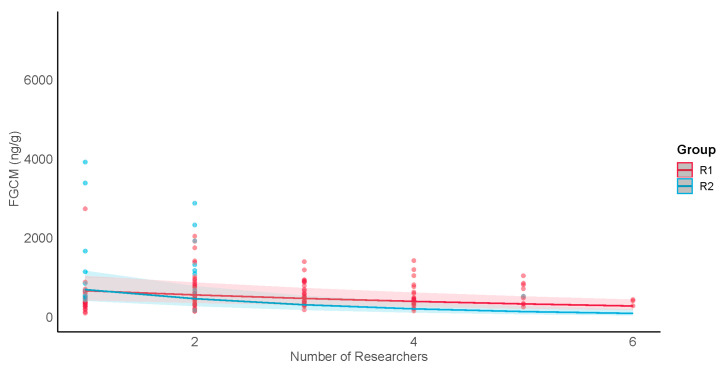
Exposure samples: male FGCM (ng/g) response to daily numbers of researchers present. Lines show predicted values for each group; shading shows 95% confidence intervals; dots show raw data points.

For the exposure tourism condition, we found a significant main effect of numbers of researchers and a significant interaction between the group and numbers of researchers for males but not females; the male FGCM levels were lower when more researchers were present in the group, and R2 levels decreased more quickly than R1 levels (Figure 6). Females showed no significant main effect or interaction with group (Figure S2).

### 3.4. Possible Effects of Sex Hormones, Diet, Physical Activity, and Environmental Conditions

In H4, we examined whether any relationships between FGCMs and human exposure that we found above could be attributed in part to variations in sex hormones, diet, physical activity, and environmental conditions. The results for models H1–H3 included fixed effects for sex hormones and the food availability index (Table 5, Table 6, Table 7, Table 8 and Table 9). In general, both sex hormones and food availability were significantly positively associated with FGCM levels. Since these variables also served as controls when examining the relationships between FGCM levels and exposure to humans, the significant results we found cannot be due wholly to relationships between FGCM levels, sex hormones, and food availability. To examine whether variations in physical activity or rainfall could explain relationships between FGCM levels and exposure to humans, we used separate GLMM models to examine whether the mean FGCM levels for individual macaques each month were related to their monthly locomotion rates, monthly rainfall, and/or the monthly number of tourists. We found significant relationships between undisturbed FGCM levels and both rainfall and locomotion for both males and females (females—rainfall: X = 8.13, *p* = 0.017; females—physical activity: X = 16.95, *p* < 0.001; males—rainfall: X = 13.16, *p* < 0.001; males—physical activity: X = 22.46, *p* < 0.001). Nevertheless, significant relationships between FGCM levels and monthly numbers of tourists were sustained for both males and females (females: X = 35.99, *p* < 0.001; males: X = 39.48, *p* < 0.001), suggesting that these relationships cannot be due wholly to relationships between FGCM levels, rainfall, or locomotion. Notably, our results were also independent from several other factors entered as control variables (Table 5, Table 6, Table 7, Table 8 and Table 9). FGCM levels were generally lower in higher-ranking subjects, in months with more crop defense, and in females when more infants were present, but were inconsistent in males with regard to the numbers of estrous females present.

## 4. Discussion

This study aimed to test the general hypothesis that glucocorticoid output in wild *M. nigra* in Tangkoko Nature Reserve, NE Sulawesi, Indonesia is related to aspects of tourism. We collected fecal samples for the measurement of glucocorticoid metabolite (FGCM) levels from three habituated social groups with varying levels of exposure to tourism. Overall, our results suggest that the two groups habituated for tourism, but not the research-only group, exhibited increased levels of FGCMs as the number of tourists in the forest increased, even though tourists were not present within the study groups. When tourists were in the tourism groups, males also showed higher levels of FGCMs on those days when many tourists were present within the group. While female FGCM levels did not apparently respond to variations in the daily numbers of tourists present within the group, those in one tourism group showed a temporal pattern of FGCM increases and decreases to the presence of any tourists that were consistent with indications of acute stress; fecal FGCMs increased from undisturbed levels 36–48 h after exposure and then returned to undisturbed levels the next day. In contrast, males in the tourism groups showed no significant differences between undisturbed, exposure, or postexposure conditions, suggesting little evidence of an FGCM response. FGCM responses to the number of researchers in the group varied by sex, group, and tourist condition. Overall, the relationships we found between FGCM responses and exposure to tourists were independent of relationships between FGCM levels and sex hormones, food availability, physical activity, and rainfall, as well as other factors we controlled (dominance rank, the frequency of crop defense, and the numbers of infants and estrous females present). As such, our findings cannot be attributed to increased metabolic demand from these sources. Thus, we tentatively suggest that tourism in Tangkoko Nature Reserve is a source of acute anthropogenic stress, but not chronic stress for some *M. nigra*, despite their being exposed to tourism for decades. Below, we develop this argument in greater detail.

(H1) In those months in which greater numbers of tourists were present in the forest, we saw increases in FGCM levels in both males and females in the tourism groups, suggesting that for tourism-habituated groups, greater numbers of tourists in the forest may increase metabolic demand, at least in the short term (H1a–c). In contrast, in the research-only group, females showed no change, and surprisingly, males showed decreased levels. These results are consistent with findings in Yucatan black howler (*Alouatta pigra)* [87] and in *G.g. gorilla* [11] that showed that tourism-exposed groups tended to have higher FGCM levels than research-only groups. Although the results for our tourism groups were consistent across groups and sexes, it should be noted that the number of undisturbed samples for these groups was modest, given that on most days, the groups experienced tourism and/or crop defense. This was especially the case for R2. While slopes for the tourist groups show marked differences from the slopes for PB1 (females: R2 = 4.0; R1 = 5.0; PB1 = 0; males: R2 = 23.3; R1 = 0.5; PB1 = −0.1), those for R2 in particular should be interpreted cautiously. A follow-up study could collect more undisturbed samples over a longer time period from the two tourism groups to better understand their responses.

It is unclear why males in the research-only group showed decreased FGCM levels in months with more tourists in the forest. Given that stress increases with uncertainty [88] and decreases when situations become more predictable [89], it may be that males in the research-only group, which was generally farther from most of the tourism activity, could detect the presence of tourists in the forest from a greater distance than the tourism groups and hence avoid their presence more easily, doing so more often when they detected more tourists in the forest. We observed the research-only group moving away from tourists during accidental encounters, likely to areas farther away from tourists than the other tourism groups. The fact that FGCM levels in females in the research-only group remained constant suggests that they may also have benefited from staying out of the areas perceived to be risky. We previously found that the same groups of *M. nigra* responded to tourists in the forest with behavioral inhibition, suggesting increased vigilance (similar to when primates detect predators at a distance) rather than increases in stress-related behavior [56]. Prior research has shown that low levels of predation risk in a variety of species may increase vigilance, without increasing FGCMs, or may even be accompanied by a decrease (see review by [90]). See also the discussion of H3 below.

(H2) When tourists were within the two tourism groups, females in R1 experienced a pattern of change in FGCM levels that was consistent with predictions for acute stress; FGCM levels rose from undisturbed levels following exposure to tourists within their groups and then returned to undisturbed levels thereafter (H2a). This result is also consistent with our earlier findings that these females displayed increases in aggression and decreases in sociality when tourists were present within their groups [56]. A return to undisturbed FGCM levels after exposure to a stressor is generally considered an adaptive response and is often seen as an indication that the stressor is unlikely to cause long-term physiological harm [91]. While this may be the case for these females, we cannot rule out the possibility that acute stress may be damaging physiologically if it occurs frequently, depending on the reproductive state of the female. Seminal research by Moberg [92,93] showed how the stress-induced secretion of cortisol can affect fertility by disrupting the synthesis and secretion of both the follicle-stimulating hormone and the luteinizing hormone. R2 females also showed a significant increase from the undisturbed to the exposure samples, but no significant decrease from exposure to postexposure samples at least within a day. This may be indicative of an inability to return quickly to undisturbed levels after experiencing a stressor (H2b). Although a prolonged return to undisturbed levels can indicate a chronic stress response, we think this is unlikely because subsequent undisturbed samples in this group were often collected after only one more additional day without tourists or crop defense. Hence, it is likely that these females returned to undisturbed levels after about two days postexposure. Males on the other hand showed no significant change and no differences in overall undisturbed FGCM levels (i.e., when not considering numbers of tourists in the forest) from males in the research-only group, results that point to a lack of a stress response to the presence of tourists (H2c) rather than chronic stress. Notably, however, despite the lack of significant differences between tourist conditions, the pattern of changes between conditions for R2 males appeared similar to that for R2 females (Figure 2a,b), raising the possibility that a larger sample size for R2 males may have yielded a similar significant pattern, and hence a similar possible inability to return quickly to undisturbed levels. Given that R2 males also returned to undisturbed levels often only a day or two after the postexposure samples, it is also unlikely that their responses can be characterized as chronic stress responses. Assuming these interpretations are correct, differences between R1 and R2 may be due to the fact that R2 experienced more frequent tourist visits and may have experienced somewhat more stress as a result.

Our findings for males where undisturbed and exposure levels both increased in those months with more tourists in the forest (H1a) and on days with more tourists within groups (H2d) could be seen as inconsistent with our findings of no significant changes from undisturbed to exposure levels (H2c); however, the fact that undisturbed levels and exposure levels to tourists within groups were similar to each other over the months suggests that these males may have been able to cope with increases in tourist numbers in a healthy manner. Similar increases in females’ FGCM levels in months with more tourists in the forest could be seen as inconsistent with R1 females’ quick recoveries from the presence of tourists within groups. However, the fact that R1 female responses to tourists within groups appeared to recover quickly, and R2 females likely returned to undisturbed levels in another day or so, also suggests that, even if tourists are a source of stress, they may also be able to cope with tourist presence in a healthy manner.

Our findings related to the nature of FGCM responses to tourism are similar to those for *P.p morio* subject to tourism. Muehlenbein et al. [26] found acute but not chronic increases in fecal cortisol metabolites after tourist visits, and in fact lower FGCM levels than in a group subject to research only. Maréchal et al. [47] also found higher fecal glucocorticoids among *M. sylvanus* after tourist encounters in Gibraltar. However, this occurred specifically in samples collected after aggressive interactions with tourists [47]. Although aggressive interactions with tourists at Tangkoko Nature Reserve are rare enough to be considered almost nonexistent (D.B., personal observations), a renewed examination of FGCM responses would be warranted should this scenario change. Additionally, our finding of increasing FGCM levels in males as the daily number of tourists increased (H2d) suggests that limiting the daily number of tourists allowed in each group may alleviate potential stress responses in the future.

(H3) When no tourists were present (i.e., during undisturbed conditions), FGCM levels increased with the numbers of researchers for females from the research-only group and for both sexes in one tourism group (R1) (H3). This is consistent with previous research showing that females in tourism groups displayed higher levels of in-group aggression on the days when more researchers were present [56]. In contrast, FGCM levels in the research-only group decreased with the numbers of researchers for males. These differences suggest that more researchers may increase metabolic demand in some groups/sexes but decrease it in others. The reason for this is unclear. Although researchers (who wore identical shirts and received rigorous training to ensure that they behaved in similar ways with the macaques) were easily distinguished from tourists, it may be that researcher gender or time spent with MNP working with these groups specifically may have played a role in our results. A more robust sample size would allow for a finer-tuned understanding of how researcher presence relates to *M. nigra* FGCM levels. Nevertheless, it may be beneficial to take a conservative approach and limit the daily number of researchers in the tourism groups to four, even on days when there are no tourists present, and to reduce the number of researchers present in the research-only group.

When tourists were present within groups (i.e., during exposure conditions), males in both tourism groups showed decreased FGCM levels as the numbers of researchers increased, suggesting that researchers may act as buffers to challenges from exposure to tourists. In contrast, females showed no response to different numbers of researchers present. Why females did not respond like males is not clear; however, it may be that a larger sample size is needed.

While there is a paucity of research specifically exploring the FGCM responses of primates to researchers, one such study focused on South African samangos (*Cercopithecus albogularis*). LaBarge et al. [94] found that female FGCM acute responses to predators flattened as observer numbers increased. While it was not possible to determine if this decreased response was due to observers inadvertently deterring predators, it does lend support to the idea that the presence of several familiar humans might affect primate perception of danger [94]. Future studies that pair FGCM levels with specific tourist/macaque interaction data may clarify this issue.

(H4) Although FGCM levels can be influenced by many internal and environmental factors [33,39,40,41,42,43,44,45,46], we attempted to control for as many such potential confounds. Not surprisingly, we found significant positive relationships between FGCM levels, sex hormones, food availability, physical activity, and rainfall, and significant generally negative associations with dominance rank, crop defense, and infant presence. Nevertheless, these relationships were unable to explain the significant relationships we found between FGCM levels and tourists, adding to the strength of our findings and highlighting the importance of controlling for as many factors as possible.

## 5. Conclusions

The present study shows little evidence of long-term harm from tourism at Tangkoko Nature Reserve. However, the story may not be complete. There are some limitations to this study. For example, we did not examine exposure samples taken after more than four consecutive days of exposure to tourists. Hence, we cannot rule out the possibility that different and more concerning patterns might appear after longer runs of days with tourists. Future studies with larger sample sizes would be helpful. Moreover, we were unable to monitor the length of time it took for exposure FGCM levels to return to undisturbed levels if it did not occur within a day. Another limitation of this study was the lack of a true control group, i.e., an unhabituated group (*cf* [62]); the research-only group encountered tourists on rare occasions. This type of limitation is common when collecting data on wild primates. Nevertheless, this study highlights the notion that important information about possible tourist effects on adrenocortical activity can be gleaned from the comparison of FGCM levels in the different groups characterized by substantial differences in tourist exposure. Additionally, although the measurement of fecal cortisol metabolites is a useful way to measure the mean levels of FGCM over a 24–36 h period, adding salivary sampling to monitor cortisol levels in the wild (*cf* [95,96]) to specific events would enable a more refined assessment of the effects of specific tourism conditions. Finally, a longer study in which feces could be sampled more frequently is needed to match samples for tourism conditions on consecutive days, and hence examine day-to-day changes in metabolic demand. While few can understate the benefits of effective primate tourism to local economies and ecosystems [97,98,99,100], the present study, along with our previous findings that these macaques display some increases in conspecific aggression in response to tourists [56], suggests that some primates may not fully habituate to tourism even after decades of exposure. Although we found no evidence of chronic stress, we believe that the effects of tourists and researchers warrant future monitoring and deeper exploration. Specifically, we suggest further research to identify the optimal number of both researchers and tourists allowed in each group each day. A comprehensive study would carefully pair each individual’s behavior, saliva sample taken under specific conditions, and daily fecal samples to uncover the ideal daily number of tourists and researchers for each group.

## Figures and Tables

**Table 1:** Hypotheses.

**Hypothesis (H) 1.** *Possible Physiological Effects of Tourists in the Forest.*
**(H1).** *If exposure to tourists increases metabolic demand (i.e., results in a general stress response), even when tourists are only in the forest, then we will find significant differences in FGCM levels that are related to levels of exposure to tourism.*
**(H1a).** *If temporal variation in tourist presence in the forest is associated with increased metabolic demand, we will find significantly higher levels of FGCM in those months with more tourists in the forest.*
**(H1b).** *If study groups with substantial tourism exposure experience higher metabolic demand than the group with little tourism exposure, then the two macaque groups regularly exposed to tourists (R1 and R2) will have higher FGCM levels than the group rarely exposed to tourists (PB1).*
**(H1c).** *If the monthly numbers of tourists in the forest affect individual study groups differently, there will be a significant interaction effect between group and numbers of tourists per month. Group responses may be related to their levels of exposure to tourism. For example, R2 (the group with the highest exposure) may have the highest increases in FGCM, R1 (the group with medium exposure) may have a less intense increase, and PB1 (the group with the lowest exposure) may have the smallest increase as the number of tourists in the forest increase. Alternatively, each group may display qualitatively different responses as the number of tourists in the forest increases.*
**Hypothesis 2.** *Possible Physiological Effects of Tourists in the Group.*
**(H2).** *If exposure to tourists within the group increases metabolic demand, then we will find significant differences in FGCM levels that are related to the presence of tourists.*
**(H2a).** *If patterns of metabolic demand are consistent with those described for acute stress in other species (see Introduction,* Section 1.1 *), then we will find moderate undisturbed FGCM levels, and increased FGCM levels during exposure that subsequently decrease in the postexposure context (returning to near undisturbed levels).*
**(H2b).** *If the patterns of metabolic demand are consistent with those described for chronic stress, then we will find relatively high undisturbed FGCM levels, but no differences between undisturbed, exposure, and postexposure levels and/or an apparent delayed decrease in the poststressor period.*
**(H2c).** *If the patterns of metabolic demand are consistent with the absence of a stress response to the presence of tourists, then we will find relatively low undisturbed levels and no differences between undisturbed, exposure, and postexposure conditions.*
**(H2d).** *If the macaques respond more when more tourists are present in the group each day than when fewer are present, then we will find higher FGCM levels in those exposure samples where more daily tourists are present.*
**Hypothesis 3.** *Possible Physiological Effects of Researchers in the Group.*
**(H3).** *If the number of researchers within study groups at once influences metabolic demand, then we will find significantly different FGCM levels when exposed to different numbers of researchers in both undisturbed and exposure conditions.*
**Hypothesis 4.** *Sex Hormones, Diet, Physical Activity, and Environment.*
**(H4).** *Finally, to shed light on whether any relationships we find between FGCM and exposure to tourism could be attributed at least in part to variations in sex hormones, diet, physical activity, or environment (rainfall), we examined the relationships between FGCM levels and tourism-related variables while controlling for sex hormones (i.e., fecal estrogen and testosterone metabolite levels in females and males, respectively), food availability, locomotion, and monthly rainfall.*

**Table 2:** Sample sizes (fecal samples) used in statistical analysis.

**Group R1**			
Macaque ID	Undisturbed	Exposure	Postexposure
cu †	3	7	2
fu †	2	10	3
gs †	3	8	4
hs †	1	11	3
nu †	3	6	2
qs †	2	2	2
**Total Female**	**14**	**44**	**16**
ak	8	33	15
ej	1	9	2
kn	8	21	5
ll	3	24	8
mm	3	22	7
om	3	14	1
**Total Male**	**26**	**123**	**38**
***Total Group***	***40***	***167***	***54***
**Group R2**			
Macaque ID	Undisturbed	Exposure	Postexposure
fd †	1	6	1
od †	2	2	1
qd †	2	3	1
id †	1	6	1
td †	1	1	1
zd †	1	1	1
**Total Female**	**8**	**19**	**6**
an	1	3	2
rm	1	2	3
rn	1	5	3
tl	1	6	1
wj	1	4	3
**Total Male**	**5**	**20**	**12**
***Total Group***	***13***	***39***	***18***
**Group PB1**			
Macaque ID	Undisturbed		
aa †	14		
ba †	13		
bp †	24		
cp †	16		
rp †	16		
up †	24		
**Total Female**	**107**		
fm	30		
ql	9		
uk	39		
ul	34		
**Total Male**	**112**		
***Total Group***	***219***		
Mean ± samples per focal subject = 13.82 ± 0.99			
**Total Fecal Samples Analyzed = 456**			

† denotes female.

**Table 3:** Fruit availability index at Tangkoko Nature Reserve 2014–2015.

FAI = (∑_1_/N) × (∑_2_/X)	
∑_1_	Sum of log food scores
∑_2_	Sum of plots (20)
N	# trees measured
X	# of trees sampled in each species in all plots
∑_1_/N	Log mean food abundance
∑_2_/X	Mean density

**Table 4:** Description of model factors *.

Factor	Definition	Variable Type	Hypothesis
FGCM level	Measure of glucocorticoid metabolite levels ng/g in a fecal sample	Response	All
Social group	R1 (tourism), R2 (tourism), and PB1 (research only)	Fixed effect	1, 2, 3, 4 *
No. tourists in forest	Total number of tourists present in the forest each month	Fixed effect	1, 2 *, 4
No. tourists within group each day	Number of tourists in the focal subject’s group on the day of sample collection	Fixed effect	2d, 3
No. tourists within group each month	Number of tourists in the focal subject’s group in the month of sample collection	Fixed effect	2 *, 3 *
No. researchers within group each day	Number of researchers present in the focal subject’s group on day of sample collection	Fixed effect	1, 3, 4 *
Tourism condition	Fecal sample represents baseline, exposure, or post-tourism conditions	Fixed effect	2a–c
Physical activity	% point time samples locomoting per month	Fixed effect	4
Rainfall	Amount of rainfall per month	Fixed effect	4
Dominance rank	David scores (females); standardized elo ratings (males)	Control factor	All
Testosterone level (males)	Testosterone level ng/g in focal male’s fecal sample	Control factor	All
Estradiol level (females)	Estradiol level ng/g in focal female’s fecal sample	Control factor	All
No. crop defense events each month	Total number of days with crop defense events each month	Control factor	All
No. estrous females each day	Number of estrous females present that day	Control factor	All
No. infants each month	Number of infants (<1 yr) present each month	Control factor	All
FAI rank	Fruit availability index ranked	Control factor	All
Monkey ID	Focal subject ID	Random effect	All
Collection time of fecal sample	Time of day the sample was collected	Random effect	All
Sample number	Sample number to control for overdispersion in one male model	Random effect	2a–c

* Control variable for particular hypotheses.

**Table 5:** Hypothesis 1a–c—relationships between undisturbed FGCM levels and numbers of tourists in the forest each month.

Males				Females			
Fixed Effects	SE	z	F	Fixed Effects	SE	z	F
No. tourists in forest	0.02	−1.44		No. tourists in forest	0.03	1.29	
Social Group			† 2.33	Social Group			† 28.04 ***
PB vs. R1	0.38	1.52		PB vs. R1	0.80	5.05 ***	
PB vs. R2	0.85	0.20		PB vs. R2	0.40	0.63	
R1 vs. R2	0.85	−0.49		R1 vs. R2	0.86	−4.44 ***	
Social Group x No. tourists in forest			† 39.48 ***	Social Group x No. tourists in forest			† 35.99 ***
PB vs. R1	0.03	−6.10 ***		PB vs. R1	0.13	−6.17 ***	
PB vs. R2	0.31	−1.85		PB vs. R2	0.21	0.81	
R1 vs. R2	0.31	−1.22		R1 vs. R2	0.26	2.41 *	
No. crop defense events each month	0.01	−3.05 **		No. crop defense events each month	0.01	−6.65 ***	
Testosterone level x Dominance rank	0.06	−5.48 ***		Estradiol level x Dominance rank	0.01	−1.80	
Testosterone level	0.04	19.14 ***		Estradiol level	0.08	4.09 ***	
Dominance rank	0.10	9.96 ***		Dominance rank	0.05	0.07	
No. estrous females each day	0.05	7.07 ***		No. infants each month	0.06	1.69	
FAI rank	0.04	4.28 ***		FAI rank	0.07	2.55 *	

*** *p* ≤ 0.001, ** *p* ≤ 0.01, * *p* ≤ 0.05; † Chisq coefficient was used to show overall significance of the interactions including social group.

**Table 6:** Hypothesis 2a–c—FGCM response to undisturbed, exposure, and postexposure tourist conditions.

Males				Females			
Fixed Effects	SE	z	F	Fixed Effects	SE	z	F
Tourism Condition		† 0.80		Tourism Condition		† 6.69 ***	
Undis vs. Exp †	0.09	0.13		Undis vs. Exp †	0.17	−2.16 **	
Undis vs. Post †	0.10	0.72		Undis vs. Post †	0.23	0.05	
Exp vs. Post †	0.08	0.83		Exp vs. Post †	0.20	1.93	
No. tourists within group each month	0.01	1.25		No. tourists within group each month	0.11	−1.34	
Social Group R1 vs. Social Group R2	0.21	0.94		Social Group R1 vs. Social Group R2	0.63	3.24 **	
Social Group x Tourism Condition			† 8.58 *	Social Group x Tourism Condition			† 18.47 ***
Undis vs. Exp †	0.22	−0.66		Undis vs. Exp †	0.47	−2.70 **	
Undis vs. Post †	0.24	−2.40 *		Undis vs. Post †	0.40	−4.26 ***	
Exp vs. Post †	0.43	−2.60 **		Exp vs. Post †	0.44	−1.34	
No. crop defense events each month	0.04	−0.70		No. crop defense events each month	0.01	−2.00 *	
Testosterone level x Dominance rank	0.04	−0.57		Estradiol level x Dominance rank	0.05	−0.15	
Testosterone level	0.04	15.08 ***		Estradiol level	0.80	1.11	
Dominance rank	0.06	1.09		Dominance rank	0.06	−2.59 *	
No. estrous females each day	0.03	1.24		No. infants each month	0.05	−3.90 **	
FAI rank	0.02	1.95		FAI rank	0.04	1.72	

*** *p* ≤ 0.001, ** *p* ≤ 0.01, * *p* ≤ 0.05. † Chisq coefficient was used to show overall significance of the interactions including social group.

**Table 7:** Hypothesis 2d—relationships between exposure FGCM levels and numbers of tourists in the group.

Males				Females			
Fixed Effects	SE	z	F	Fixed Effects	SE	z	F
No. tourists within group each day	0.00	6.37 ***		No. tourists within group each day	0.01	0.03	
No. tourists within group each month	0.01	2.51 *		No. tourists within group each month	0.00	−2.85 **	
Social Group R1 vs. Social Group R2	0.42	1.17		Social Group R1 vs. Social Group R2	0.34	2.88 **	
Social Group x No. tourists within group each month			† 25.67 ***	Social Group x No. tourists within group each month			† 3.63
R1 vs. R2	0.00	5.06 ***		R1 vs. R2	0.00	−1.91	
Social Group x No. tourists within group each day			† 25.77 ***	Social Group x No. tourists within group each day			† 2.02
R1 vs. R2	0.01	−5.08 ***		R1 vs. R2	0.01	1.42	
No. crop defense events each month	0.03	−2.92 **		No. crop defense events each month	0.02	−0.53	
Testosterone level x Dominance rank	0.04	1.57		Estradiol level x Dominance rank	0.03	−1.32	
Testosterone level	0.03	14.49 ***		Estradiol level	0.50	2.29 *	
Dominance rank	0.06	−3.07 **		Dominance rank	0.02	−5.85 **	
No. estrous females each day	0.02	−6.13 ***		No. infants each month	0.06	−4.61 ***	
FAI rank	0.04	4.14 ***		FAI rank	0.05	−1.52	

*** *p* ≤ 0.001, ** *p* ≤ 0.01, * *p* ≤ 0.05; † Chisq coefficient was used to show overall significance of the interactions including social group.

**Table 8:** Hypothesis 3a—relationships between FGCM levels and numbers of researchers (undisturbed samples).

Undisturbed Samples							
Males				Females			
Fixed Effects	SE	z	F	Fixed Effects	SE	z	F
No. researchers within group each day	0.02	−1.40		No. researchers within group each day	0.04	0.46	
Social Group			† 16.08 ***	Social Group			† 8.80 *
PB vs. R1	0.44	1.39		PB vs. R1	0.34	2.95 **	
PB vs. R2	0.45	−2.72 **		PB vs. R2	0.27	−0.97	
R1 vs. R2	0.45	−2.84 **		R1 vs. R2	0.38	−1.95	
Social Group x No. researchers within group each day			† 23.61 ***	Social Group x No. researchers within group each day			† 22.27 ***
PB vs. R1	0.06	−4.85 ***		PB vs. R1	0.07	−4.72 ***	
PB vs. R2	na	na		PB vs. R2	0.22	−0.56	
R1 vs. R2	na	na		R1 vs. R2	0.22	0.85	
No. crop defense events each month	0.03	−3.39 ***		No. crop defense events each month	0.01	−9.13 ***	
Testosterone level x Dominance rank	0.08	−3.04 **		Estradiol level x Dominance rank	0.01	0.12	
Testosterone level	0.05	14.86 ***		Estradiol level	0.07	3.70 ***	
Dominance rank	0.11	8.76 ***		Dominance rank	0.03	−0.10	
No. estrous females each day	0.07	4.87 ***		No. infants each month	0.04	−1.35	
FAI rank	0.03	7.01 ***		FAI rank	0.05	4.02 ***	

*** *p* ≤ 0.001, ** *p* ≤ 0.01, * *p* ≤ 0.05; † Chisq coefficient was used to show overall significance of the interactions including social group.

**Table 9:** Hypothesis 3b—relationships between FGCM levels and numbers of researchers (exposure samples).

Exposure Samples							
Males				Females			
Fixed Effects	SE	z	F	Fixed Effects	SE	z	F
No. researchers within group each day	0.02	−10.76 ***		No. researchers within group each day	0.07	−1.94	
No. tourists within group each month	0.00	−4.85 ***		No. tourists within group each month	0.00	−3.97 ***	
Social Group R1 vs. Social Group R2	0.36	−1.56		Social Group R1 vs. Social Group R2	0.74	1.49	
Social Group x No. tourists within group each month			† 25.67 **	Social Group x No. tourists within group each month			† 8.48 **
R1 vs. R2	0.00	5.08 **		R1 vs. R2	0.00	−2.91 **	
Social Group x No. researchers within group each day			† 10.62 **	Social Group x No. researchers within group each day			† 1.73
R1 vs. R2	0.07	3.26 **		R1 vs. R2	0.20	−2.06 *	
No. crop defense events each month	0.02	−9.97 ***		No. crop defense events each month	n/a	n/a	
Testosterone level x Dominance rank	0.04	5.36 **		Estradiol level x Dominance rank	0.03	−0.53	
Testosterone level	0.03	16.97 ***		Estradiol level	0.47	1.59	
Dominance rank	0.05	−7.71 ***		Dominance rank	0.02	−7.57 ***	
No. estrous females each day	0.02	−8.78 ***		No. infants each month	0.05	−5.97 ***	
FAI rank	0.03	4.13 ***		FAI rank	0.07	−2.39 *	

*** *p* ≤ 0.001, ** *p* ≤ 0.01, * *p* ≤ 0.05; † Chisq coefficient was used to show overall significance of the interactions including social group.

## Data Availability

Data are available upon request to the first author and main contact.

## Supplementary Materials

The following supporting information can be downloaded at: https://www.mdpi.com/article/10.3390/ani13182842/s1, Figure S1. Female FGCM (ng/g) response to daily numbers of tourist present within the group. Lines show predicted values for each group; shading shows 95% confidence intervals; dots show raw data points. Figure S2. Female FGCM (ng/g) response to daily numbers of researchers present within the group. Lines show predicted values for each group; shading shows 95% confidence intervals; dots show raw data points. Table S1. *Macaca nigra* Focal Subjects. Table S2. *Macaca nigra* Behaviors Analyzed. Table S3. Detailed Description of Model Factors.
