# Supplementary material for: Effects of Tourist and Researcher Presence on Fecal Glucocorticoid Metabolite Levels in Wild, Habituated Sulawesi Crested Macaques (Macaca nigra)
Source: Animals (Basel). 2023 Sep 7;13(18):2842. doi: 10.3390/ani13182842 (PMC10525950; doi:10.3390/ani13182842)

Figure S1. Female FGCM (ng/g) response to daily numbers of tourist present within the group. Lines show predicted values for each group; shading shows 95% confidence intervals; dots show raw data points.

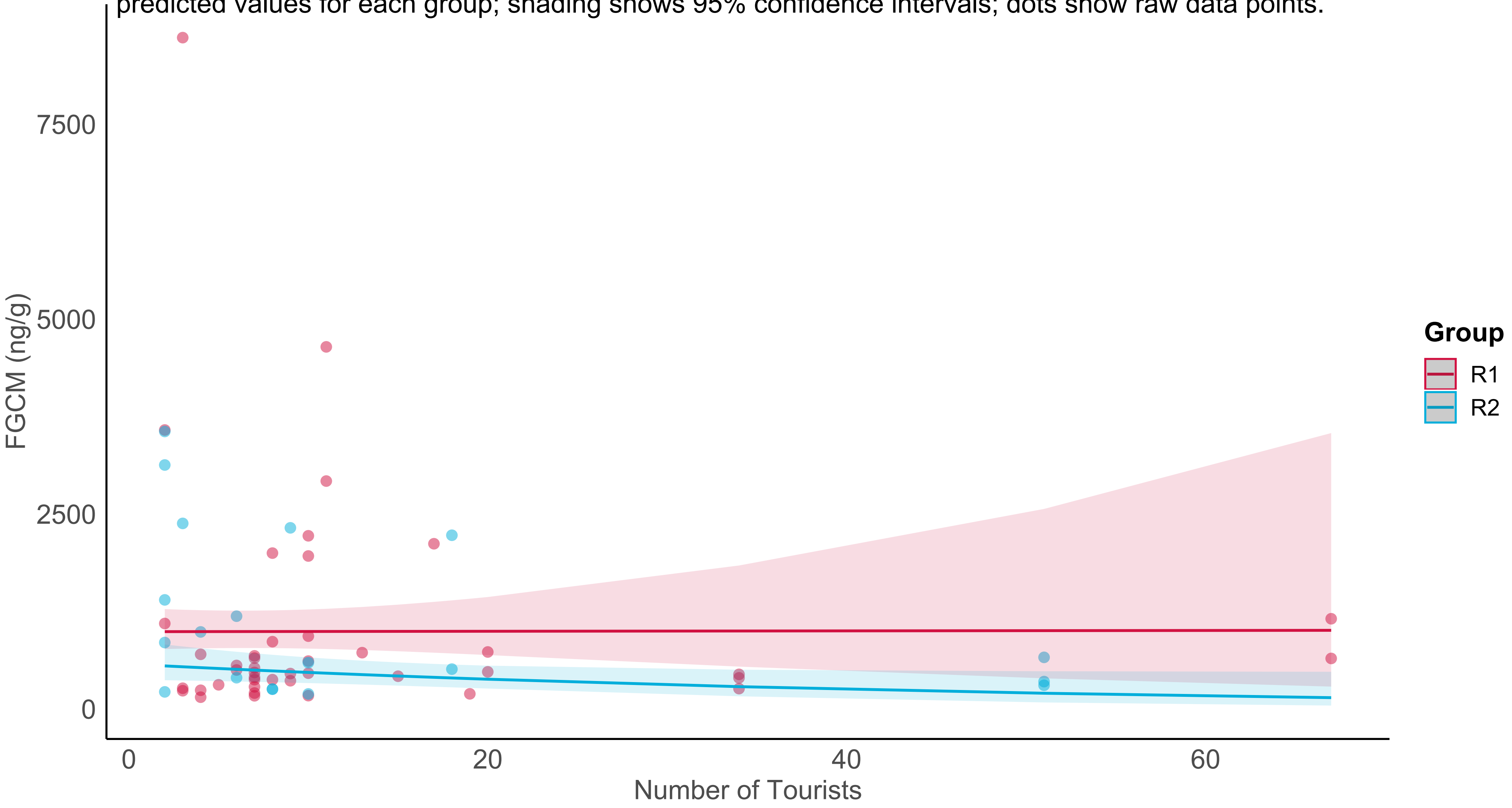

Supplement: Supplementary file 1 [file animals-13-02842-s001.zip › Figure S1 COLOR Female FGCM ng-g Response to Daily Tourists.pdf]
