# Supplementary material for: Effects of Tourist and Researcher Presence on Fecal Glucocorticoid Metabolite Levels in Wild, Habituated Sulawesi Crested Macaques (Macaca nigra)
Source: Animals (Basel). 2023 Sep 7;13(18):2842. doi: 10.3390/ani13182842 (PMC10525950; doi:10.3390/ani13182842)
Supplement: Supplementary file 1 [file animals-13-02842-s001.zip › Table S2 - TourismFecalTangkokoEthogramofMacaca_nigraBehaviors.pdf]

Table S2. *Macaca nigra* Behaviors Analyzed[1]

BEHAVIOR	DEFINTION
**PHYSICAL ACTIVITY – Point Time Sampling**	
**walk**	Actively moving at a slow pace.
**run**	Actively moving at a quick pace.
**climb up**	Actively climbing up.
**climb down**	Actively climbing down.
**AGONISTIC-- Continuous Sampling**	
**chase**	To run after another individual on more than 2 meters to make it run away and/or bite/hit/grab it.
**bite**	To bite another individual.
**hit**	To hit another individual with any limb
**grab**	To catch and hold in a hand a bunch of fur of another one to retain it.
**push**	To push with hand or body to make an individual moving away.
**lunge**	To make a short run (< 2 meters) or a jump toward an individual (warning that could lead to an aggressive behavior.)
**scream**	Noisy scream vocalization.
**flight**	To run away in response to another's approach/aggression.
**crouch**	To press body on the ground, 4 limbs flexed in response to another's approach/aggression.
**protection seeking**	An individual threatened or attacked by another approaches and contacts a third individual.

[1] Definitions were based on those used by the Macaca Nigra Project. A few were modified to fit the needs of this study.
